# A keyword searchable attribute-based encryption scheme with attribute update for cloud storage

**DOI:** 10.1371/journal.pone.0197318

**Published:** 2018-05-24

**Authors:** Shangping Wang, Jian Ye, Yaling Zhang

**Affiliations:** 1 School of Science, Xi’an University of Technology, Xi’an, Shaanxi, China; 2 School of Computer Science and Engineering, Xi’an University of Technology, Xi’an, Shaanxi, China; University of Texas at San Antonio, UNITED STATES

## Abstract

Ciphertext-policy attribute-based encryption (CP-ABE) scheme is a new type of data encryption primitive, which is very suitable for data cloud storage for its fine-grained access control. Keyword-based searchable encryption scheme enables users to quickly find interesting data stored in the cloud server without revealing any information of the searched keywords. In this work, we provide a keyword searchable attribute-based encryption scheme with attribute update for cloud storage, which is a combination of attribute-based encryption scheme and keyword searchable encryption scheme. The new scheme supports the user's attribute update, especially in our new scheme when a user's attribute need to be updated, only the user's secret key related with the attribute need to be updated, while other user's secret key and the ciphertexts related with this attribute need not to be updated with the help of the cloud server. In addition, we outsource the operation with high computation cost to cloud server to reduce the user's computational burden. Moreover, our scheme is proven to be semantic security against chosen ciphertext-policy and chosen plaintext attack in the general bilinear group model. And our scheme is also proven to be semantic security against chosen keyword attack under bilinear Diffie-Hellman (BDH) assumption.

## Introduction

Attribute-based encryption (ABE)[1–4] is regarded as an effective encryption method with fine grained access control in the cloud storage. Attribute-based encryption can be divided into two types of key-policy attribute-based encryption [1] (KP-ABE) and ciphertext-policy attribute-based encryption [2] (CP-ABE). The KP-ABE scheme refers to that the ciphertext is associated with an attribute set, and a user's secret key is associated with an access policy. A user can decrypt the ciphertext if and only if the ciphertext's attribute set satisfy the access policy of user’s secret key. The CP-ABE scheme refers to that the ciphertext is associated with an access policy, and a user's secret key is associated with an attribute set. A user is can decrypt the ciphertext if and only if his attribute set satisfy the access policy of the ciphertext.

At present, many ABE schemes [5–9] have been proposed, which provide secure data access control and overcome the shortcomings of one-to-one encryption pattern in identity-based encryption scheme. However, these schemes are still defective to be used in practice, as the attribute of a user is dynamic, which may be changed over time. Thus the attribute revocation mechanism is necessary for ABE scheme to be used in practice.

The revocation mechanism can be divided into two types: direct revocation mechanism and indirect revocation mechanism. Imai and Attrapaduang [10] gives a clear definition of direct revocation and indirect revocation. Direct revocation is defined as: the sender specifies a revocation list when encrypting the data. Indirect revocation is defined as: the authorized institutions regularly issue key updates to non-revoked users. At present, many schemes with direct revocation [11–14] have been proposed. Li et al. [11] proposed an identity-based revocation scheme that performs directed revocation by giving the revocation rights to encipherer directly. Tu et al. [14] proposed a revocable ABE scheme. In addition, some indirect attribute revocation schemes[15–18] have also been proposed. Yu et al.[15] proposed an attribute based data sharing scheme with attribute revocation. In this scheme, user’s any attribute can be revoked by proxy re-encryption technique. Li et al. [18] proposed a scheme that supports user’s attribute revocation, but the scheme could only revoke a single attribute of the user, thus it could not satisfy the actual needs.

The attribute update is another significant problem in the ABE environment. In actual life, a user's attribute set may need to be updated over time when his working role may be changed. For example, assume that Alice is a company employee, then her attribute set needs to be updated when her working role is promoted from a programmer lifted to a project manager, thus her former attribute set A = "female, programmer" should be changed to a new attribute set B = "female, project manager". And the attribute authority (AA) should issue an update key to update Alice's secret key. Meanwhile, the attribute authority must ensure that the employee Alice cannot further use her previous key related to the attribute set "female, programmer" to access the ciphertexts. Thus, the attribute update is not a simple process. Some attribute update schemes [19–21]have been proposed. However, these schemes have a common problem, the problem is that if there is a user's an attribute is updated, and then many other user's secret key and a lot of ciphertexts related with this attribute need to be updated, which will undoubtedly waste a lot of computational resources.

To address this problem, we give a feasible solution in this paper. The main idea of our solution is that the secret key of a user is divided into two parts, one part which is irrelevant to attribute is retained by the user, and the other part which is relevant to attribute is sent to the cloud server(CS). When an attribute of any user needs to be updated, the AA issues an update key to CS. Then CS only updates the secret key of this attribute for all valid users, and other secret key of all user and the ciphertexts related with this attribute need not to be updated. This method will greatly reduce the work load of the system.

Although attribute based encryption technology provides an effective means for data confidentiality, yet it brings another new problem that the users may find it difficult to search for interesting data from a vast number of encrypted data. This problem is called keyword search problem [22]. One of the simplest searching methods is to download all encrypted data locally and then to decrypt it, finally to execute keyword search in plaintext. However, this method will waste huge computational resource and bring a vast cost for user to do the work of decryption.

Another extreme searching method is to send the secret key of the user and keywords to CS, then CS decrypts all of the ciphertexts and performs searching operation on plaintext. But this method will expose the user's secret key and privacy of search keyword to CS, this is infeasible. Some search-based encryption schemes [23–26] have been proposed. Such as Boneh et al. [23] first proposed a public key encryption with keyword search scheme. Dan and Ostrovsky[24] proposed a public key cryptographic scheme that allows privacy data retrieval (PIR), and allows multiple data contributors to upload their data with public key by encryption algorithm, and only the user with the corresponding secret key can decrypt the data.

Some search encryption schemes [27–30] focuses on search efficiency have also been proposed. Fu et al. [27] proposed a scheme that not only supports multi-keyword ranked search but also provides parallel search. Li et al. [28] put forward a scheme which supports multi-keyword search. In this scheme, users can retrieve multiple keywords at once, and which greatly improves the search efficiency and search accuracy. Sun et al. [30] proposed a verifiable attribute-based keyword search scheme that supports fine-grained search authorization scheme. In this scheme, multiple data owners and multiple users are supported, and the scheme also supports fine-grained search authorization.

In addition, some schemes focus on achieving both attribute revocation and keyword search have been proposed[31,32].The schemes[31,32]which not only support user’s multiple attributes revocation but also provide keyword search. However, our new scheme is different from the schemes [31,32], and the differences between our scheme and schemes [31,32]can be described as follows: firstly, the scheme[32] is based on the key-policy(KP-ABE). The new scheme and the scheme [31] are based on the ciphertext policy (CP-ABE), where the scheme [31]makes use of the access tree as access policy, while the new scheme makes use of LSSS as access policy. So the new scheme is different from the schemes [31,32]. Secondly, the scheme [31] supports public keyword searchable, and the keyword index and trapdoor are generated with the help of the cloud server. The ciphertext and the keyword index of the scheme [32] are associated with attribute. The new scheme also supports public key keyword search, but the keyword index and trapdoor generation phase is independently realized by user. Furthermore, the based on the difficult problems of the new scheme and schemes [31,32]are different. The scheme [31] is proven to be secure under the assumption of bilinear Diffie-Hellman (BDH)in selective security model. The scheme [32] is proven to be secure under the assumption of decisional bilinear Diffie-Hellman exponent(q-BDHE) and decisional Diffie-Hellman (DDH) in the selective security model. The new scheme is proven to be secure in the general bilinear group model.

### Our contributions

In this paper, we propose a keyword searchable attribute-based encryption scheme with attribute update for cloud storage. The main contributions of our scheme are summarized as follows:

1. The new scheme is a combination of ABE scheme and keyword searchable encryption scheme. So our scheme not only solves the problem of confidentiality of the data with fine -grained access control but also solves the problem of keyword search. Moreover, the scheme is proven to be semantic security against chosen ciphertext-policy and chosen plaintext attack in the general bilinear group model.
2. The new scheme supports the user's attribute update, and when a user’s attribute need to be updated, only the user's secret key related with this attribute need to be updated, while other users’ secret key and the ciphertexts related with the attribute need not to be updated. This is a more efficient attribute update method than that in existing attribute update schemes.
3. In addition, the operation with high computation cost is outsourced to CS to reduce the user's computational burden.
4. Our keyword search algorithm supports multi-user keywords searchable, as long as user's trapdoor could match keywords index stored in the cloud storage. Moreover, our keyword search scheme is proved to be semantic security against chosen keyword attack (IND-CKA) under bilinear Diffie-Hellman (BDH) assumption.

### Functional comparisons

We compare the function of our scheme with some exiting schemes [13,19,21,29,31] in Table 1.

**Table 1:** The comparisons of our scheme with some exiting schemes.

schemes	access control	secret key update for update user	No secret key update for non-update user	keyword searchable	No ciphertext update
[13]	LSSS	✓	✘	✘	✘
[19]	access tree	✓	✘	✘	✘
[21]	access tree	✓	✘	✘	✘
[29]	LSSS	✓	✘	✘	✘
[31]	access tree	✓	✘	✓	✘
Ours	LSSS	✓	✓	✓	✓

✘: there is no corresponding function in the scheme.

✓: there is corresponding function in the scheme.

## Preliminaries

### Bilinear map [33]

Let $G_{0}$ and $G_{1}$ be two multiplicative cyclic bilinear groups of prime order *p*. Let *g* be a generator of $G_{0}$. A bilinear map is a map $e:\;G_{0}\times G_{0}\to G_{1}$ with the following properties:

1. Bilinearity: for all $g\in G_{0}$ and $a,b\in Z_{p}$, we have *e*(*g*^*a*^,*g*^*b*^) = *e*(*g*,*g*)^*ab*^.
2. Non-degeneracy: *e*(*g*,*g*) ≠ 1.
3. Computability: There is an efficient algorithm to compute *e*(*u*,*v*) for $u,v\in G_{0}$.

### Bilinear Diffie-Hellman assumption [34]

The BDH problem in $G_{0}$ is defined as follows: taken $(g,g^{a},g^{b},g^{c})\in G_{0}$ as input, compute $e(g,g{)}^{abc}\in G_{1}$. We say that the adversary A has *ε* advantage in solving BDH problems in $G_{0}$ if

$$Pr|[A(g,g^{a},g^{b},g^{c})=e(g,g{)}^{abc}]|\geq \varepsilon$$

We say that the BDH assumption holds in $G_{0}$ if no probability polynomial adversary A has non-negligible advantage in solving the BDH problem in $G_{0}$.

### Generic bilinear group model [2]

We suppose there are two random encodings $\Psi_{0},\Psi_{1}:\;Z_{p}^{+}\to \{0,1{\}}^{*}$, where $Z_{p}$ is an additive group and m > 3*logp*. For *i* = 0,1, we set $G_{i}=\{\Psi_{i}(x):x\in Z_{p}^{+}\}$. We are given oracles to compute the induced group action on $G_{0},G_{1}$ and an oracle to compute a non-degenerate bilinear map $e:\;G_{0}\times G_{0}\to G_{1}$. And we are also given a random oracle to represent the hash function *H*.

### Linear secret sharing schemes [33]

A linear secret sharing scheme ∏ over a set of parties P is called linear (over $Z_{p}$) if

1. The shares for each party form a vector over $Z_{p}$.
2. There exists a matrix **M** with *l* rows and *n* columns called the share-generating matrix for ∏. For all *i* = 1,2,⋯,*l*, the function *ρ* defines the party labeling ith row of **M** as *ρ*(*i*). When we consider the column vector $v=(s,r_{2},\cdots ,r_{n})\in Z_{p}^{n}$, where $s\in Z_{p}$ is the secret to be shared, and $r_{2},\cdots ,r_{n}\in Z_{p}^{n}$ are randomly chosen. Then **M***v* is the vector of 1shares of the secret *s* according to ∏. The share (**M***v*)_***i***_ belongs to party *ρ*(*i*).

Suppose ∏ that is an LSSS for the access structure A. Let S∈A be any authorized set, and *I* ⊂ {1,⋯,*l*}. Then, there exist constants ${\left\{\omega_{i}\in Z_{p}\right\}}_{i\in I}$ such that, if {*λ*_*i*_} are valid shares of any secret *s* according to ∏, then ∑_*i*∈I_ *ω*_*i*_ *λ*_*i*_ = *s*. Furthermore,there these constants {*ω*_*i*_} can be found in time polynomial in the size of the share -generating matrix **M**.

## System model and security model

### System model

**Fig 1:**
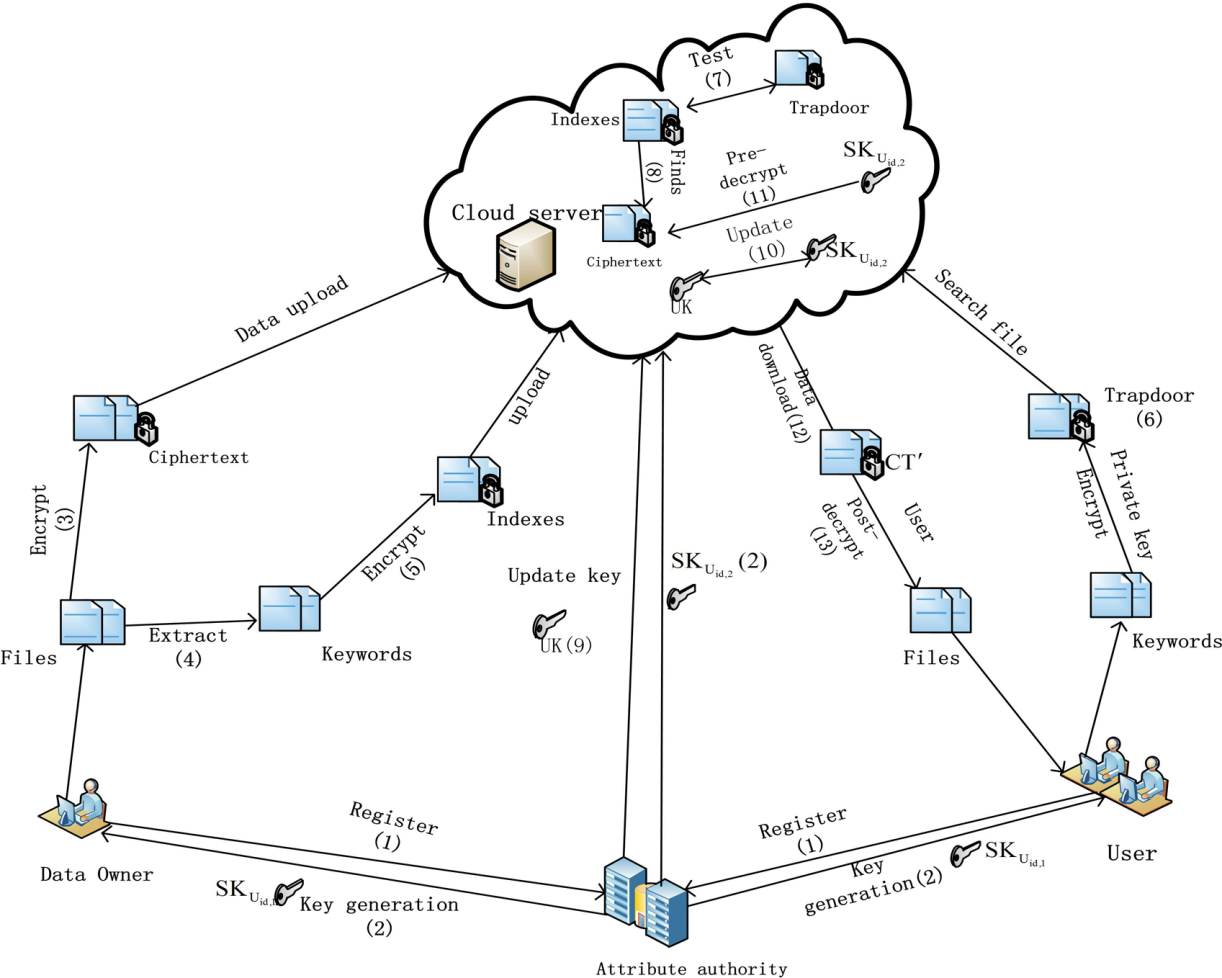
System model of the proposed scheme.

A system framework of our scheme includes the main four entities is presented in Fig 1.

**Attribute authority (AA).** The AA is a perfectly trusted entity. It takes charge of the system establishment, user registration, attributes management and secret key generation. And when an attribute of a user needs to be updated, the AA generates an updated key for the user.

**Cloud server (CS).** The CS is responsible for storing the data and providing data access for legitimate users. It is also responsible for keyword search when a search trapdoor is received from a user. And it also takes charge of updating the user's partial secret key which related to the updated attribute and helps legitimate users to partially decrypt the ciphertext by using partial secret key of the user.

**Data owner (DO).** The data owner encrypts its owner data and builds keyword indexes, and then outsources them to the CS.

**User (U).** Each legitimate user can search their interesting the files from system. The user generates a search trapdoor to protect the privacy of the search keyword. Then the user sends his identity and search trapdoor to CS. Without revealing any information about keyword search, the CS will find the encrypted file includes the keywords and do a lot of partial decryption work to reduce the decryption load of the user. Finally, the user gets the partial decrypted files, and then decrypts the partial decrypted files by using his owner partial secret key.

### Algorithm description

We proposed a keyword searchable attribute-based encryption scheme with attribute update for cloud storage includes the following eight phases.

#### Phase 1: System initialization

**AA.Setup** (*λ*,*L*) → (*PP*,*MSK*,*PK*_*s*_,*SK*_*s*_). The setup algorithm inputs a security parameters λ and an attribute universe *L*, and outputs the public parameters *PP*, the master secret key *MSK*, the CS's public and secret key pairs (*PK*_*s*_,*SK*_*s*_).

#### Phase 2: Key generation

**AA.KeyGen** $\left(MSK,id,S_{U_{id}})\to ({SK}_{U_{id,1}}{,SK}_{U_{id,2}},X_{U_{id}},A_{priv},B_{pub}\right)$. The key generation algorithm inputs the master secret key *MSK*, an user's identity *id* and the user's attribute set $S_{U_{id}}$, and outputs the user's secret key $\left({SK}_{U_{id,1}}{,SK}_{U_{id,2}},X_{U_{id}}\right)$, the user's search secret and public key pairs (*A*_*priv*_,*B*_*pub*_).

#### Phase 3: File encryption and create keyword index

To get ciphertext *E*_*k*_(*F*), the DO encrypts file *F* with symmetric key *k* by the symmetric encryption algorithm. Then DO encrypts the symmetric key *k* by the following encryption algorithm.

**DO.Encrypt** (*PP*,*k*,(**M**,*ρ*)) → *CT*. The encryption algorithm inputs the public parameter *PP*, the symmetric key *k* and the LSSS access structure (M,*ρ*), and outputs a ciphertext *CT*.

**DO.Index** (*W*,*B*_*pub*_) → *I*_*W*_. The index generation algorithm inputs a set of keywords *W* and data owner's search public key *B*_*pub*_, and outputs the keywords index set *I*_*W*_.

#### Phase 4: Trapdoor generation

**U.AuthorizationKey** $(PP,{PK}_{s},X_{U_{id}})\to K_{U_{id}}^{\prime }$. The authentication information generation algorithm inputs public parameters *PP*, the CS's public key *PK*_*s*_ and the user's secret key $X_{U_{id}}$, and outputs the authentication information $K_{U_{id}}^{\prime }$.

**U.Trapdoor** (*w*,*PK*_*s*_,*A*_*priv*_) → *T*_*w*_. The trapdoor generation algorithm inputs a keyword *w*, the CS's public key *PK*_*s*_ and the user's search secret key *A*_*priv*_, and outputs the search trapdoor *T*_*w*_.

#### Phase 5: Verification

**CS.Verifing** $\left(id,K_{U_{id}}^{\prime })\to (0,1\right)$. The validation algorithm inputs user's identity *id* and the authentication information $K_{U_{id}}^{\prime }$, and outputs 1or 0.

#### Phase 6: File retrieval

**CS.Test** (*I*_*W*_,*T*_*w*_) → (0,1). The test algorithm inputs the keywords index set *I*_*W*_ and the user's search trapdoor *T*_*w*_, and outputs1or 0.

#### Phase 7: Data decryption

**CS.PreDecrypt** $(CT,{SK}_{U_{id,2}})\to {CT}^{\prime }$. The pre-decryption algorithm inputs the ciphertext *CT* for the access structure (**M**,*ρ*) and user's secret key ${SK}_{U_{id,2}}$ for the attribute set $S_{U_{id}}$. If the user's attribute set $S_{U_{id}}$ satisfies the access structure (**M**,*ρ*). It outputs a partial decrypted ciphertext *CT*′. Otherwise, the algorithm is terminated.

**U.PostDecrypt** $({CT}^{\prime },{SK}_{U_{id,1}})\to k$. The post-decryption algorithm inputs the partial decrypted ciphertext *CT*′ and the user's secret key ${SK}_{U_{id,1}}$, and outputs symmetric key *k*.

Finally, the user decrypts the file *E*_*k*_(*F*) by the symmetric key *k*, and then the user gets the file *F*.

#### Phase 8: Attribute update

Assume that a user with identity *id* whose an attribute ${j\in S}_{U_{id}}$ needs to be updated to a new attribute *j*′ by the AA. The attribute update phase includes five steps: (1) The AA executes update key algorithm to generate update key ${UK}_{j\to j^{\prime }}$ and sends it to CS, and the AA informs CS that the user with identity *id* and his an attribute *j* will be updated to a new attribute *j*′; (2)The CS finds user's attribute set $S_{U_{id}}$ and secret key ${SK}_{U_{id,2}}$ in the SL-list; (3)The CS updates the attribute *j* of the user to the attribute *j*′, and sets the new attribute set as $S_{U_{id}}≔\{S_{U_{id}}\backslash \{j\}\}\cup \{j^{\prime }\}$; (4) The CS also updates secret key $\left\{j:D_{j},D_{j}^{\prime }\right\}$ associates with attribute *j* to new secret key $\left\{j^{\prime }:D_{j^{\prime }},D_{j^{\prime }}^{\prime }\right\}$ associates with attribute *j*′ by update key ${UK}_{j\to j^{\prime }}$; (5) The CS retains user's new attribute set $S_{U_{id}}$ and new secret key ${SK}_{U_{id,2}}^{\prime }$ in the SL-list.

**AA.UKeyGen** $(PP,MSK,j,j^{\prime })\to {UK}_{j\to j^{\prime }}$. The update key generation algorithm inputs the public parameter *PP*, the master secret key *MSK*, the attribute *j* and *j*′, and outputs the update key ${UK}_{j\to j^{\prime }}$.

**CS.KeyUpdate** $({SK}_{U_{id,2}},{UK}_{j\to j^{\prime }})\to {SK}_{U_{id,2}}^{\prime }$. The secret key update algorithm inputs user's secret key ${SK}_{U_{id,2}}$ and the update key ${UK}_{j\to j^{\prime }}$, and outputs a new secret key ${SK}_{U_{id,2}}^{\prime }$.

### Security model

#### (1) Selective security model for our scheme

**Initialization.** The adversary A submits a challenged access structure $A^{*}$ to the challenger C.

**Setup.** The challenger C runs the setup algorithm and sends the public parameters *PP* to the adversary A and keeps the master key *MSK* to itself.

**Phase 1**. The adversary A adaptively issues repeated secret keys corresponding to attribute sets *S*_1_,*S*_2_⋯*S*_*q*_, where none of these attribute sets satisfy the access structure $A^{*}$.

**Challenge**. The adversary A submits two equal-length messages *M*_0_ and *M*_1_ to C. The challenger C randomly selects a bit *b* ∈ {0,1} and encrypts the message *M*_*b*_ for the access structure $A^{*}$. The challenger C sends the ciphertext CT^*^ to the adversary A.

**Phase 2.** Phase1 is repeated.

**Guess.** The adversary A outputs a guess *b*′ of *b*. If *b*′ = *b*, the adversary A wins this game.

The advantage of the adversaryA in this game is defined as $Adv=\left|Pr(b^{\prime }=b)-\frac{1}{2}\right|$.

**Definition 1.** The proposed scheme is selective security if all polynomial time adversaries have at most a negligible advantage in the above game.

#### (2) IND-CKA security model

**Setup**. Repeat the above security model 's setup.

**Phase1**. The adversary A adaptively issues polynomial following queries.

***H***_**1**_**,*H***_**2**_**-Query.** The adversary A can query the random oracle *H*_1_ or *H*_2_.

**Trapdoor Queries**. The adversary A can query any keywords trapdoor.

**Challenge.** The adversary A submits two keywords *w*_0_ and *w*_1_ to the challenger C, with the restriction that the adversary A has not queried the trapdoors of keywords *w*_0_ and *w*_1_. The challenger C randomly chooses a bit *b* ∈ {0,1} and generates the index *I*_*b*_ of keyword *w*_*b*_.

**Phase 2.** Phase1 is repeated.

**Guess**. The adversary A outputs a guess *b*′ of *b*. If *b*′ = *b*, the adversary A wins this game.

The advantage of the adversaryA in this game is defined as $Adv=\left|Pr(b^{\prime }=b)-\frac{1}{2}\right|$.

**Definition 2**. The proposed scheme is IND-CKA secure if all polynomial time adversaries have at most a negligible advantage in the above game.

## Concrete construction

In this section, we present a construction for a keyword searchable attribute-based encryption scheme with attribute update for cloud storage.

### Phase 1: System initialization

The AA first defines an attribute universe as *L* = {1,2,⋯,*m*} and chooses three hash functions $H:{\left\{0,1\right\}}^{*}\to G_{0},H_{1}:{\left\{0,1\right\}}^{*}\to G_{0}$ and $H_{2}:G_{1}\to \{0,1{\}}^{logp}$, which can be modeled as random oracles. Then the CS creates a user identity list $SL=(id,S_{U_{id}},{SK}_{U_{id,2}},K_{U_{id}})$ and a file list FL = (F^*^,CT,*I*_*W*_,*E*_*k*_(*F*)), which are initially empty. Finally, the AA executes the setup algorithm.

**AA.Setup** (*λ*,*L*) → (*PP*,*MSK*,*PK*_*s*_,*SK*_*s*_). The setup algorithm first chooses two multiplicative cyclic groups $G_{0}$ and $G_{1}$ of prime order *p*, a generator ${g\in G}_{0}$ and a bilinear map $e:G_{0}\times G_{0}\to G_{1}$. It then chooses $x_{s}\in Z_{p}$ and lets *SK*_*s*_ = *x*_*s*_ as the CS's secret key. And it computes ${PK}_{s}=g^{x_{s}}$ as the CS's public key and publishes it. And it also randomly chooses three elements $\alpha ,\gamma ,\mu \in Z_{p}$. In addition, it chooses a random number $v_{j}\in Z_{p}$ for each attribute *j* ∈ *L*. Finally, it outputs the public parameters *PP* and the master secret key *MSK* as follows:

$$PP=\{G_{0},G_{1},g,g^{\gamma },g^{\mu },e(g,g{)}^{\alpha },\{{PK}_{j}=H(j{)}^{v_{j}},{PK}_{j}^{\prime }=g^{v_{j}}|j\in L\}\}$$

$$MSK=\{\gamma ,\mu ,g^{\alpha },\{v_{j}|j\in L\}\}$$

### Phase 2: Key generation

The AA first distributes an attribute set $S_{U_{id}}\subseteq L$ associates with user's identity id, when a user with identity *id* requests a registration in the system. Secondly, the AA randomly chooses a number $X_{U_{id}}\in Z_{p}$ for the user and calculates $K_{U_{id}}=g^{\frac{\gamma }{X_{U_{id}}}}$. Then, the AA executes key generation algorithm.

**AA.KeyGen** $\left(MSK,id,S_{U_{id}})\to ({SK}_{U_{id,1}}{,SK}_{U_{id,2}},X_{U_{id}},A_{priv},B_{pub}\right)$. The key generation algorithm first randomly chooses $t_{id}\in Z_{p}$, which *t*_*id*_ is a unique assigned to the user with identity *id*. For each attribute ${j\in S}_{U_{id}}$, it randomly chooses $v_{j}\in Z_{p}$. Finally, it outputs the user's secret key:

$${SK}_{U_{id,1}}=D=g^{\alpha }∙g^{t_{id}}$$

$${SK}_{U_{id,2}}=\{\forall {j\in S}_{U_{id}}:D_{j}=H(j{)}^{\frac{t_{id}}{v_{j}}},D_{j}^{\prime }=g^{\frac{t_{id}}{v_{j}}},v_{j}\in MSK\}$$

Lets *A*_*priv*_ = *μ* as the user's search secret key and *B*_*pub*_ = *g*^*μ*^ as the user's search public key.

Finally, the AA sends ${(X}_{U_{id}},{SK}_{U_{id,1}},A_{priv})$ to the user and publishes the user's searches public key *B*_*pub*_. And, the AA sends $\left(id,S_{U_{id}},{SK}_{U_{id,2}},K_{U_{id}}\right)$ to the CS, the CS stores $\left(id,S_{U_{id}},{SK}_{U_{id,2}},K_{U_{id}}\right)$ in the SL-list.

### Phase 3: File encryption and keyword index generation

Step 1: The DO encrypts the files.

To get ciphertext *E*_*k*_(*F*), the DO encrypts file *F* with a symmetric key *k* by the symmetric encryption algorithm. Then DO encrypts the symmetric key *k* by the following encryption algorithm.

**DO.Encrypt** (*PP*,*k*,(M,*ρ*)) → *CT*. Let **M** be an *l* × *n* matrix, and **M**_*i*_ be the vector corresponding to the *i* th row of matrix **M**. The function *ρ* associates rows of matrix **M** to attributes. The encryption algorithm first chooses a random vector $v=(s,y_{2},\ldots y_{n}){\in Z}_{p}^{n}$. These elements of vector **v** will be used to share the random encryption exponent *s*. For *i* = 1 to *l*, it calculates *λ*_*i*_ = M_*i*_**v**^*T*^. It then randomly chooses numbers $r_{1},\ldots ,r_{l}\in Z_{p}$ and outputs the ciphertext *CT*:

$$CT=\{(M,\rho ),\tilde{C}=k∙e(g,g{)}^{\alpha s},C=g^{s},$$

$$C_{i}=(g^{v_{\rho (i)}}{)}^{\lambda_{i}}H(\rho (i){)}^{v_{\rho (i)}r_{i}},C_{i}^{\prime }=(g^{v_{\rho (i)}}{)}^{r_{i}},i=1,2,\cdots ,l\}$$

Where *v*_*ρ*(*i*)_ refers to the master key is associated with attribute *ρ*(*i*) ∈ *L*.

Step2: Index generation.

The DO extracts a keywords set $W=(w_{1},w_{2},\cdots ,w_{n^{\prime }})$ from the file *F*. Then DO executes the following index generation algorithm.

**DO.Index** (*W*,*B*_*pub*_) → *I*_*W*_. The index generation algorithm randomly chooses $\xi_{i}{\in Z}_{p}$ for each keyword *w*_*i*_ ∈ *W* and calculates $\tau_{i}=e((B_{pub}{)}^{\xi_{i}},H_{1}(w_{i}))$. It then lets $I_{w_{i}}=[I_{1},I_{2}]=[g^{\xi_{i}},H_{2}(\tau_{i})]$. Finally, It outputs the keywords index set $I_{W}=\{I_{w_{i}}{\}}_{i\in \{1,2,\cdots ,n^{\prime }\}}$.

Finally, the DO sends the file (*CT*,*I*_*W*_,*E*_*k*_(*F*)) to the CS. When the CS receives the uploaded file (*CT*,*I*_*W*_,*E*_*k*_(*F*)), it picks a unique identifier F^*^ for the file (*CT*,*I*_*W*_,*E*_*k*_(*F*)). The CS stores (F^*^,*CT*,*I*_*W*_,*E*_*k*_(*F*)) in the FL-list.

### Phase 4: Trapdoor generation

Step 1: Authentication information generation.

**U.AuthorizationKey** $(PP,{PK}_{s},X_{U_{id}})\to K_{U_{id}}^{\prime }$. To generate the authentication information, the authentication information generation algorithm chooses a random number $\chi {\in Z}_{p}$ and calculates $K_{1}=({PK}_{s}{)}^{\chi }(g^{\gamma }{)}^{\frac{1}{X_{U_{id}}}},K_{2}=g^{\chi }$. It outputs the authentication information $K_{U_{id}}^{\prime }$:

$$K_{U_{id}}^{\prime }=\{K_{1},K_{2}\}=\{({PK}_{s}{)}^{\chi }(g^{\gamma }{)}^{\frac{1}{X_{U_{id}}}},g^{\chi }\}$$

Step 2: Trapdoor generation.

**U.Trapdoor** (*w*,*PK*_*s*_,*A*_*priv*_) → *T*_*w*_. The trapdoor generation algorithm randomly chooses a number $\eta {\in Z}_{p}$ and calculates *T*_1_ = *g*^*η*^, $T_{2}=({PK}_{s}{)}^{\eta }∙{(H}_{1}(w{))}^{A_{priv}}$. It outputs the trapdoor *T*_*w*_:

$$T_{w}=\{T_{1},T_{2}\}=\{g^{\eta },\;({PK}_{s}{)}^{\eta }∙{(H}_{1}(w{))}^{A_{priv}}\}=\{g^{\eta },(g^{x_{s}}{)}^{\eta }{(H}_{1}(w{))}^{\mu }\}\}$$

Finally, the user sends his *id*, the authentication information $K_{U_{id}}^{\prime }$ and the trapdoor *T*_*w*_ to CS.

### Phase 5:Verification

**CS.Verifing** $\left(id,K_{U_{id}}^{\prime })\to (0,1\right)$. The validation algorithm inputs user's identity *id* and the authentication information $K_{U_{id}}^{\prime }$. The cloud server uses its own secret key *SK*_*s*_ = *x*_*s*_ to calculate

$$K_{U_{id}}^{\prime \prime }=\frac{K_{1}}{(K_{2}{)}^{x_{s}}}=g^{\frac{\gamma }{X_{U_{id}}}}$$

and judges the equation $K_{U_{id}}=K_{U_{id}}^{\prime \prime }$ holds or not. If the equation holds, which means the user is a legitimate user, it outputs 1. Otherwise, it outputs 0 and the algorithm is terminated.

### Phase 6: File retrieval

**CS.Test** (*I*_*W*_,*T*_*w*_) → (0,1). The test algorithm inputs the keywords index set *I*_*W*_ and the user's search trapdoor *T*_*w*_. The cloud server uses its own secret key *SK*_*s*_ = *x*_*s*_ and user's trapdoor *T*_*w*_ = {*T*_1_,*T*_2_} to calculate

$$\varphi =\frac{T_{2}}{(T_{1}{)}^{x_{s}}}=\frac{({PK}_{s}{)}^{\eta }∙{(H}_{1}{\left(w)\right)}^{A_{priv}}}{(g^{\eta }{)}^{x_{s}}}=\frac{\left(g^{x_{s}}{)}^{\eta }∙H_{1}^{\mu }(w\right)}{(g^{\eta }{)}^{x_{s}}}=H_{1}^{\mu }(w)$$

It then accords to keywords index $I_{w_{i}}=[I_{1},I_{2}]=[g^{\xi_{i}},H_{2}(\tau_{i})]$ to calculate

$$\varphi_{1}=e(I_{1},\varphi )=e(g^{\xi_{i}},H_{1}^{\mu }(w))=e((g^{\mu }{)}^{\xi_{i}},H_{1}(w))$$

and judges the equation *H*_2_(*φ*_1_) = *I*_2_ holds or not. If the equation holds, which means the test is successful it outputs 1. Otherwise, it outputs 0 and the algorithm is terminated.

### Phase 7: Data decryption

Step 1: Partial decryption by CS.

The CS first obtains ciphertext *CT* corresponding to keywords index *I*_*W*_ in the FL-list and finds user's secret key ${SK}_{U_{id,2}}$ in the SL-list. If user's secret key ${SK}_{U_{id,2}}$ is not the SL-list, the algorithm ends. Otherwise, it executes pre-decryption algorithm.

**CS.PreDecrypt** $(CT,{SK}_{U_{id,2}})\to {CT}^{\prime }$. The pre-decryption algorithm inputs user's secret key ${SK}_{U_{id,2}}$ for an attribute set $S_{U_{id}}$ and a ciphertexts *CT* for access structure (**M**,*ρ*). At present, we assume that the attribute set $S_{U_{id}}$ satisfies the access structure (**M**,*ρ*) and let *I* be defined as $I=\{i|\rho (i)\in S_{U_{id}}\}$. Then, let $\{\omega_{i}\in Z_{p}{\}}_{i\in I}$ be as set of constants such that, if {*λ*_*i*_}_*i*∈I_ are valid shares of the secret *s* according to **M**, then ∑_*i*∈I_ *ω*_*i*_*λ*_*i*_ = *s*. The pre-decryption algorithm calculates

$$\begin{array}{l} A=\frac{\prod_{i\in I}e{\left(C_{i},D_{\rho \left(i\right)}^{\prime }\right)}^{\omega_{i}}}{\prod_{i\in I}e{\left(C_{i}^{\prime },D_{\rho \left(i\right)}\right)}^{\omega_{i}}}\\ \;=\frac{\prod_{i\in I}e{\left({\left(g^{v_{\rho \left(i\right)}}\right)}^{\lambda_{i}}H{\left(\rho \left(i\right)\right)}^{v_{\rho \left(i\right)}r_{i}},g^{\frac{t_{id}}{v_{\rho \left(i\right)}}}\right)}^{\omega_{i}}}{\prod_{i\in I}e{\left({\left(g^{v_{\rho \left(i\right)}}\right)}^{r_{i}},H{\left(\rho \left(i\right)\right)}^{\frac{t_{id}}{v_{\rho \left(i\right)}}}\right)}^{\omega_{i}}}\\ \;=\frac{\prod_{i\in I}e{\left({\left(g^{v_{\rho \left(i\right)}}\right)}^{\lambda_{i}},g^{\frac{t_{id}}{v_{\rho \left(i\right)}}}\right)}^{\omega_{i}}\cdot e{\left(H{\left(\rho \left(i\right)\right)}^{v_{\rho \left(i\right)}r_{i}},g^{\frac{t_{id}}{v_{\rho \left(i\right)}}}\right)}^{\omega_{i}}}{\prod_{i\in I}e{\left({\left(g^{v_{\rho \left(i\right)}}\right)}^{r_{i}},H{\left(\rho \left(i\right)\right)}^{\frac{t_{id}}{v_{\rho \left(i\right)}}}\right)}^{\omega_{i}}}\\ \;=\frac{\prod_{i\in I}e{\left(g^{\lambda_{i}},g^{t_{id}}\right)}^{\omega_{i}}\prod_{i\in I}e{\left(H{\left(\rho \left(i\right)\right)}^{r_{i}},g^{t_{id}}\right)}^{\omega_{i}}}{\prod_{i\in I}e{\left(g^{r_{i}},H{\left(\rho \left(i\right)\right)}^{t_{id}}\right)}^{\omega_{i}}}\\ \;=e{\left(g,g^{t_{id}}\right)}^{\sum_{i\in I}\omega_{i}\lambda_{i}}\\ \;=e{\left(g,g\right)}^{t_{id}s} \end{array}$$

Finally, The CS sends part-ciphertext ${CT}^{\prime }=(\tilde{C,}C,A)$ and the encrypted file *E*_*k*_(*F*) to the user.

Step2: User decryption

**U.PostDecrypt** $({CT}^{\prime },{SK}_{U_{id,1}})\to k$. The post-decryption algorithm inputs the partial decrypted ciphertext *CT*′ and the user's secret key ${SK}_{U_{id,1}}$. The user executes post-decryption algorithm to calculate symmetric *k* as follows:

$$k=\frac{\tilde{C}∙{CT}^{\prime }}{e(C,D)}=\frac{k∙e(g,g{)}^{\alpha s}∙e(g,g{)}^{t_{id}s}}{e(g^{s},g^{\alpha }∙g^{t_{id}})}=\frac{k∙e(g,g{)}^{\alpha s}∙e(g,g{)}^{t_{id}s}}{e(g,g{)}^{\alpha s}∙e(g,g{)}^{t_{id}s}}$$

Finally, the user gets the plaintext *F* = *D*_*k*_(*E*_*k*_(*F*)) by the symmetric key *k*.

### Phase 8: Attribute update

Step1: Update key generation.

**AA.UKeyGen** $(PP,MSK,j,j^{\prime })\to {UK}_{j\to j^{\prime }}$. The update key generation algorithm inputs the public parameter *PP*, the master secret key *MSK*, the attribute *j* and *j*′. For attribute *j*′, the AA finds the random number $v_{j^{\prime }}\in Z_{p}$ in the master secret key *MSK*, and then the AA outputs the update key ${UK}_{j\to j^{\prime }}$:

$${UK}_{j\to j^{\prime }}=\{{UK}_{j\to j^{\prime }}^{\prime }=H(j{)}^{\frac{-t_{id}}{v_{j}}}∙H(j^{\prime }{)}^{\frac{t_{id}}{v_{j^{\prime }}}},{UK}_{j\to j^{\prime }}^{\prime \prime }=\frac{v_{j}}{v_{j^{\prime }}}\}$$

Finally, it sends user's identity *id* and update key ${UK}_{j\to j^{\prime }}$ to CS.

Step2:The CS executes a secret key update.

**CS.KeyUpdate** $({SK}_{U_{id,2}},{UK}_{j\to j^{\prime }})\to {SK}_{U_{id,2}}^{\prime }$. The secret key update algorithm inputs user's secret key ${SK}_{U_{id,2}}$ and the update key ${UK}_{j\to j^{\prime }}$. The CS executes the secret key update algorithm, and outputs a new secret key ${SK}_{U_{id,2}}^{\prime }$.

$${SK}_{U_{id,2}}^{\prime }=\{\forall {i\in S}_{U_{id}}\backslash \{j\}:D_{i}=H(i{)}^{\frac{t_{id}}{v_{i}}},D_{i}^{\prime }=g^{\frac{t_{id}}{v_{i}}}$$

$$D_{j^{\prime }}=H(j{)}^{\frac{t_{id}}{v_{j}}}∙{UK}_{j\to j^{\prime }}^{\prime },D_{j^{\prime }}^{\prime }=g^{\frac{t_{id}}{v_{j}}.{UK}_{j\to j^{\prime }}^{\prime \prime }}\}$$

## Security analysis

### Selective security proof for our scheme

**Theorem 1.** Let $\psi_{0},\psi_{1},G_{0}$ and $G_{1}$ be defined as in the generic bilinear group model. For any adversary A that makes a total of at most *q* queries to the oracles for computing the group operations in $G_{0}$ and $G_{1}$, the bilinear map *e* and the interaction with the IND-sCP-CPA security game, then the advantage of the adversary A in the IND-sCP-CPA security game is O(q2p).

**Proof.** In the IND-sCP-CPA security game, the challenge ciphertext has part-ciphertext $\tilde{C}$ may be *k*_0_*e*(*g*,*g*)^*αs*^ or *k*_1_*e*(*g*,*g*)^*αs*^. As in the [2], we modify ciphertext in the IND-sCP-CPA security game, now assuming the challenge ciphertext $\tilde{C}$ which may be *e*(*g*,*g*)^*αs*^ or *e*(*g*,*g*)^*θ*^, where $\theta \in Z_{p}$ is randomly selected and the adversary A needs to determine which is the case. Obviously, any adversary A has advantage *ε* in the IND-sCP-CPA security game may be converted into A has at least $\frac{\varepsilon }{2}$ advantage in the modified IND-sCP-CPA security game (there are two situations can be considered: one in which the adversary A must distinguish between *k*_0_*e*(*g*,*g*)^*αs*^ and *e*(*g*,*g*)^*θ*^; another in which the adversary A must distinguish between *k*_1_*e*(*g*,*g*)^*αs*^ and *e*(*g*,*g*)^*θ*^. Obviously, both of these are equivalent to the above modified IND-sCP-CPA security game).

**Initialization.** The adversary A first submits an access structure (M^*^,ρ^*^) to the simulator *S*. In order to simulate the modified IND-sCP-CPA game, and then we introduce some mathematical symbols in the general bilinear group model, and let *ψ*_0_(0) = *g*,*ψ*_1_(1) = *e*(*g*,*g*) (we will write *ψ*_0_(*x*) = *g*^*x*^,*ψ*_1_(*y*) = *e*(*g*,*g*)^*y*^).

**Setup**. The simulator *S* randomly chooses $\alpha ,\gamma ,\mu \in Z_{p}$, and calculates *g*^*μ*^,*g*^*γ*^,*e*(*g*,*g*)^*α*^. When the adversary A queries hash value of *H* on any attribute *j*, if it did not be queried, the simulator *S* randomly chooses $x_{j}\in Z_{p}$, and then calculates $H(j)=g^{x_{j}}$, and writes the results into the Hash list. Otherwise, it looks for the Hash list. For any attribute *j* ∈ *L*, the simulator *S* randomly chooses a number $v_{j}\in Z_{p}$. It sets the public parameter *PP* and the master secret key *MSK* as:

$$PP=\{g^{\gamma },g^{\mu },e(g,g{)}^{\alpha },\{{PK}_{j}=H(j{)}^{v_{j}}=g^{{x_{j}v}_{j}},{PK}_{j}^{\prime }=g^{v_{j}}|j\in L\}\}$$

$$MSK=\{\gamma ,\mu ,g^{\alpha },\{v_{j}|j\in L\}\}$$

The simulator *S* sends public parameter *PP* to the adversary A.

**Phase 1.** The simulator *S* answers secret key queries as following:

**Secret key query.** When A makes its *m*'th key generation query for the attribute set *S*_*m*_, with a constraint that attribute set *S*_*m*_ does not satisfy access structure (M^*^,ρ^*^). The simulator *S* randomly chooses $t_{m}\in Z_{p}$, and then calculates $D=g^{\alpha }∙g^{t_{m}}$. For any attribute *j* ∈ *S*_*m*_, the simulator *S* randomly chooses $v_{j}\in Z_{p}$ and calculates $D_{j}=H(j{)}^{\frac{t_{m}}{v_{j}}},D_{j}^{\prime }=g^{\frac{t_{m}}{v_{j}}}$. It outputs secret key:

$$SK=\{D=g^{\alpha }∙g^{t_{m}},\forall {j\in S}_{U_{m}}:D_{j}=H(j{)}^{\frac{t_{m}}{v_{j}}}=(g^{x_{j}}{)}^{\frac{t_{m}}{v_{j}}},D_{j}^{\prime }=g^{\frac{t_{m}}{v_{j}}}\}$$

Then, the simulator *S* sends *SK* to adversary A.

**Challenge.** The adversary A submits two equal messages *k*_0_ and *k*_1_ to the simulator *S*. First, the simulator *S* executes encryption algorithm according to the access structure (M^*^,ρ^*^). Where M^*^ is an *l* × *n* matrix. The $M_{i}^{*}$ is *i*th row of matrix M^*^. The function ρ^*^ which associates rows of matrix M^*^ to attributes. Secondly, the simulator *S* chooses a random vector $v=(s,y_{2},\ldots y_{n}){\in Z}_{p}^{n}$. These elements of vector **v** will be used to share the encryption exponent *s*. Where ${\lambda_{i}=M}_{i}^{*}v^{T}$ is constrained by the LSSS scheme. Then, the simulator *S* chooses a random variable *b* ∈ {0,1} and *l* random variable values $r_{1},\ldots ,r_{l}\in Z_{p}$ to get the encryption of $k_{b}\in G_{1}$ as: $C=g^{s},\tilde{C}=k_{b}e(g,g{)}^{\alpha s},C_{i}=g^{v_{\rho^{*}(i)}\lambda_{i}+x_{\rho^{*}(i)}v_{\rho^{*}(i)}r_{i}}$ and $C_{i}^{\prime }=g^{v_{\rho^{*}(i)}r_{i}}$.

The ciphertext is

$${CT}^{*}=\{(M^{*},\rho^{*}),C=g^{s},\tilde{C}=k_{b}e(g,g{)}^{\alpha s},$$

$$C_{i}=g^{v_{\rho^{*}(i)}\lambda_{i}+x_{\rho^{*}(i)}v_{\rho^{*}(i)}r_{i}},C_{i}^{\prime }=g^{v_{\rho^{*}(i)}r_{i}},i=1,2,\cdots ,l\}$$

Finally, the simulator *S* sends the ciphertext CT^*^ to adversary A.

**Phase 2**. Phase1 is repeated.

The adversary A terminates and returns a guess *b*′ of *b* after many queries. At this point, the simulator *S* randomly chooses a value $\theta \in Z_{p}$ to get the simulated challenge ciphertext via substituting $\tilde{C}=e(g,g{)}^{\theta }$ for $\tilde{C}=k_{b}e(g,g{)}^{\alpha s}$. After the simulation, the simulator *S* returns the simulated challenge ciphertext to adversary A.

Next, we analyze the simulator *S* simulation. We think that the simulator *S* simulation is flawless with a constraint “unexpected collision” does not occur in the querying of *ψ*_0_(*x*) = *g*^*x*^, *ψ*_1_(*y*) = *e*(*g*,*g*)^*y*^ for group operation $G_{0}$ and $G_{1}$. Thus, an “unexpected collision” occurs when two queries corresponding to two different rational functions *v* and *v*′, it causes that *v*′ − *v* = 0 for some variables. (Where an oracle query is regard as a rational function $v=\frac{\eta }{\xi }$ [2]). Then, we make the following analysis of "unexpected collision":

**Before substitution.** By the Schwartz-Zipple lemma[35,36], the probability of the “unexpected collision” occurs in $G_{0}$ and $G_{1}$ at most is O(q2p).

**After substitution.** We consider what the adversary’s view would have been if we set *θ* = *αs*. We will show that subject to the conditioning above, the the adversary’s view would have been identically distributed. Since we are in the generic group model where each group element’s representation is uniformly and independently chosen[2], the only way that the adversary’s view can differ in the case of *θ* = *αs* is if there are two queries *v* and *v*′ into $G_{1}$ is *v* ≠ *v*′ but *v*|_*θ* = *αs*_ = *v*′|_*θ* = *αs*_. We prove show that this does never happens.

**Case.** To structure *γ*′*αs*, we know that *θ* only exists as *e*(*g*,*g*)^*θ*^ in this form. According to the simulation, the simulator *S* wants *v* and *v*′ is related to the *θ* is by having some additive terms of the form *γ*′*θ*.Therefore, we must have *v* − *v*′ = *γ*′*αs* − *γ*′*θ* for some constant *γ*′ ≠ 0. Then, we artificially add the query *v* − *v*′ + *γ*′*θ* = *γ*′*αs* to the adversary's queries. According to the conditions which we have set, we prove that adversary A cannot construct the query for $e(g,g{)}^{\gamma^{\prime }\alpha s}$. Otherwise, a collision occurs and the theorem proves.

In order to gain a better understand of the above situation. We analyze based on the information given to the adversary A by the simulation. In Table 2, we enumerate the possibility queries of all rational function in $G_{1}$ by the adversary A. Except those in which every monomial involves the variable *μ*, since the variable *μ* is not relevant to constructing term *αs*. Where the variables *j* and *j*′ represents the attribute string, and *m* indicates secret key queries made by the adversary A.

**Table 2:** Possible query types from the adversary.

$x_{j}x_{j^{\prime }}$	${(v}_{\rho^{*}(i)}\lambda_{i}+x_{\rho^{*}(i)}v_{\rho^{*}(i)}r_{i})∙x_{j^{\prime }}$	${x_{j}v}_{\rho^{*}(i)}r_{i}$	${(x}_{j})(\frac{x_{j^{\prime }}t_{m}}{v_{j^{\prime }}})$
$\frac{{x_{j}t}_{m}}{v_{j^{\prime }}}$	*x*_*j*_	*γ*′*α* + *t*_*m*_	*αs + st*_*m*_
${(v}_{\rho^{*}(i)}\lambda_{i}+x_{j}v_{\rho^{*}(i)}r_{i})∙{(v}_{\rho^{*}(i^{\prime })}\lambda_{i^{\prime }}+x_{j^{\prime }}v_{\rho^{*}(i^{\prime })}r_{i^{\prime }})$	${(v}_{\rho^{*}(i^{\prime })}\lambda_{i^{\prime }}+x_{j^{\prime }}v_{\rho^{*}(i^{\prime })}r_{i^{\prime }}){∙(v}_{\rho^{*}(i)}r_{i})$	${(v}_{\rho^{*}(i^{\prime })}\lambda_{i^{\prime }}+x_{\rho^{*}(i^{\prime })}v_{\rho^{*}(i^{\prime })}r_{i^{\prime }})∙(\frac{{x_{j}t}_{m}}{v_{j}})$	${(v}_{\rho^{*}(i)}\lambda_{i}+{(x}_{\rho^{*}(i)}v_{\rho^{*}(i)}r_{i})∙(\frac{t_{m}}{v_{j}})$
$v_{\rho^{*}(i)}\lambda_{i}+x_{\rho^{*}(i)}v_{\rho^{*}(i)}r_{i}$	$v_{\rho^{*}(i)}v_{\rho^{*}(i^{\prime })}r_{i}r_{i^{\prime }}$	$\left(\frac{x_{j^{\prime }}t_{m}}{v_{j^{\prime }}})(v_{\rho^{*}(i)}r_{i}\right)$	$\left(v_{\rho^{*}(i)}r_{i})(\frac{t_{m}}{v_{j}}\right)$
$v_{\rho^{*}(i)}r_{i}$	$\left(\frac{x_{j}t_{m}}{v_{j}})(\frac{x_{j^{\prime }}t_{m}}{v_{j^{\prime }}}\right)$	$\left(\frac{{x_{j}t}_{m}}{v_{j}})(\frac{t_{m}}{v_{j}}\right)$	$\frac{{x_{j}t}_{m}}{v_{j}}$
$\left(\frac{t_{m}}{v_{j}})(\frac{t_{m}}{v_{j^{\prime }}}\right)$	tmvj	*s*	

According to Table 2, the adversary A can construct a polynomial *αs* + *t*_*m*_*s* is by pairing *s* with *α* + *t*_*m*_. In this way, the adversary A also constructs a query term containing $\gamma^{\prime }\alpha s+\sum_{m\in T}\gamma_{m}^{\prime }t_{m}s$ for some collections *T* and constant $\gamma_{m}^{\prime }\neq 0$. But the goal of the adversary A is to obtain a query polynomial *γ*′*αs*, so the adversary A must add the negative terms $\sum_{m\in T}\gamma_{m}^{\prime }t_{m}s$ to cancel the terms $\sum_{m\in T}\gamma_{m}^{\prime }t_{m}s$. To construct the negative terms $\sum_{m\in T}\gamma_{m}^{\prime }t_{m}s$, the adversary A first constructs a query polynomial of the from *t*_*m*_*s* by pairing $v_{\rho^{*}(i)}\lambda_{i}+x_{j}v_{\rho^{*}(i)}r_{i}$ with $\frac{t_{m}}{v_{j}}$ with a constraint ρ^*^(*i*) = *j*, as we know *s* is linear combinations of *λ*_*i*_. For the other collections $T_{m}^{\prime }$ and constant $\gamma_{\left(i,m,j\right)}^{\prime }\neq 0$, the adversary A can also construct a query polynomial as:

$$\gamma^{\prime }\alpha s+\sum_{m\in T}{(\gamma }_{m}^{\prime }t_{m}s+\sum_{m\in T}\gamma_{\left(i,m,j\right)}^{\prime }(v_{\rho^{*}(i)}\lambda_{i}+x_{\rho^{*}(i)}v_{\rho^{*}(i)}r_{i})∙\frac{t_{m}}{v_{j}})$$

$$=\gamma^{\prime }\alpha s+\sum_{m\in T}{(\gamma }_{m}^{\prime }t_{m}s+\sum_{m\in T}\gamma_{\left(i,m,j\right)}^{\prime }(\lambda_{i}t_{m}+x_{\rho^{*}(i)}r_{i}t_{m}))+\cdots$$

Therefore, we do some analysis to give the conclusion of this proof:

1. The set of secret shares $L_{m}=\{\lambda_{i}:\rho^{*}(i)=j,(i,j)\in T_{m}^{\prime }\}$ do not reconstruct secret *s* for some *m* ∈ *T*. Then term *t*_*m*_*s* will still be retained, and A cannot construct *γ*′*αs*.
2. If for all *m* ∈ *T* the set of secret shares $L_{m}=\{\lambda_{i}:\rho^{*}(i)=j,(i,j)\in T_{m}^{\prime }\}$ allow reconstruction the secret *s*. In order to get *γ*′*αs*, the adversary A may cancel the term $\gamma_{m}^{\prime }t_{m}s$ by the combination of the terms *t*_*m*_*λ*_*i*_, but A dose not get the term $x_{\rho^{*}(i)}t_{m}r_{i}$ by examining the Table 2, there is no term such that A can cancel this term $\gamma_{m}^{\prime }t_{m}s$. Therefore, the adversary A cannot construct *γ*′*αs*.

### IND-CKA security proof

**Theorem 2.** Supposing that BDH assumption holds, our scheme is semantically secure against a chosen keyword attack in the random oracle model.

**Proof.** Suppose the adversary A is a malicious cloud server that has non-negligible advantage *ε* in breaking our constructed searchable encryption scheme. Suppose that the adversary A makes at most $q_{H_{2}}$ hash function queries to *H*_2_ and at most *q*_*T*_ trapdoor queries(we assume $q_{H_{2}}$ and *q*_*T*_ are positive).We will construct a simulator B to solve BDH problem with advantage ε′=2ε(eqH2qT), where *e* is the base of the natural.

**Initialization.** The simulator B receives a BDH challenge and chooses two multiplicative cyclic groups $G_{0}$ and $G_{1}$ of prime order *p*, a generator ${g\in G}_{0}$ and a bilinear map $e:G_{0}\times G_{0}\to G_{1}$. Then simulator B randomly chooses $a,b,c\in Z_{p}$, lets $u_{1}=g^{a},u_{2}=g^{b},u_{3}=g^{c}\in G_{0}$, its aim is to compute $e(g,g{)}^{abc}\in G_{1}$.

**Setup.** The simulator B randomly chooses a number $x_{s}\in Z_{p}$, lets public key ${PK}_{s}=g^{x_{s}}$ and secret key *SK*_*s*_ = *x*_*s*_ for the adversary A. To simulate the user's search public keys *B*_*pub*_ and secret keys *A*_*priv*_, the simulator B chooses a random parameter $t_{1}\in Z_{p}$ and sets $B_{pub}=u_{1}^{t_{1}}$, so *A*_*priv*_ = *μ* = *at*_1_.

**Phase1**.The adversary A adaptively issues following queries:

*H*_1_**-Query:** The adversary A can query the random oracle *H*_1_ at any time. To answer to *H*_1_ queries, the simulator B maintains a list of tuples (*w*_*i*_,*h*_*i*_,*e*_*i*_,*c*_*i*_) called the *H*_1_-list. The list is initially empty. When A queries the random oracle *H*_1_ of any keywords *w*_*i*_ ∈ {0,1}^*^, the simulator B answers as follows:

1. If the query *w*_*i*_ has already appeared on the *H*_1_-list in a tuple (*w*_*i*_,*h*_*i*_,*e*_*i*_,*c*_*i*_), the simulator B responds with $H_{1}(w_{i})=h_{i}\in G_{0}$.
2. Otherwise, B generates a random coin *c*_*i*_ ∈ {0,1} so that $Pr[c_{i}=0]=\frac{1}{q_{T}+1}$, where *q*_*T*_ is a trapdoor query.

If *c*_*i*_ = 0, the simulator B calculates $h_{i}=u_{2}^{e_{i}}\in G_{0}$;

If *c*_*i*_ = 1, the simulator B calculates $h_{i}=g^{e_{i}}\in G_{0}$, where the value $e_{i}\in Z_{p}$ randomly is selected. Then B adds the tuple (*w*_*i*_,*h*_*i*_,*e*_*i*_,*c*_*i*_) to the *H*_1_-list, and returns *H*_1_(*w*_*i*_) = *h*_*i*_ to the adversary A.

*H*_2_**-Query:** A can query the random oracle *H*_2_ at any time. To answer to *H*_2_ queries, the simulator B maintains a list of tuples (*t*_*i*_,*V*_*i*_) called the *H*_2_-list. The list is initially empty. When A queries the random oracle *H*_2_ of any $t_{i}\in G_{1}$, the simulator B answers as follows:

1. If the query *t*_*i*_ has already appeared on the *H*_2_-list in a tuple (*t*_*i*_,*V*_*i*_), the simulator B responds with *H*_2_(*t*_*i*_) = *V*_*i*_ ∈ {0,1}^*logp*^.
2. Otherwise, the simulator B randomly chooses a value *V*_*i*_ ∈ {0,1}^*logp*^, and the simulator B adds the tuple (*t*_*i*_,*V*_*i*_) to the *H*_2_-list, and returns *H*_2_(*t*_*i*_) = *V*_*i*_ to A.

**Trapdoor queries:** When A queries the trapdoor of any keywords *w*_*i*_ ∈ {0,1}^*^, the simulator B first executes the *H*_1_ queries to obtain $h_{i}\in G_{0}$ such that *H*_1_(*w*_*i*_) = *h*_*i*_ and (*w*_*i*_,*h*_*i*_,*e*_*i*_,*c*_*i*_) corresponding to the tuple on the *H*_1_-list. the simulator B answers as follows:

1. If *c*_*i*_ = 0, the simulator B declares the failure and aborts.
2. If *c*_*i*_ = 1, $h_{i}=g^{e_{i}}\in G_{0}$. the simulator B randomly chooses a value $\eta \in Z_{p}$, and calculates

$$T_{1}^{*}=g^{\eta },\;T_{2}^{*}=({g^{x_{s}})}^{\eta }∙(B_{pub}{)}^{e_{i}}=({g^{x_{s}})}^{\eta }(u_{1}^{t_{1}}{)}^{e_{i}}=({PK}_{s}{)}^{\eta }H_{1}(w_{i}{)}^{\mu }$$

The simulator B sends trapdoor $T^{*}=\{T_{1}^{*},T_{2}^{*}\}$ to A.

**Challenge:** The adversary A submits a pair of keywords *w*_0_ and *w*_1_, where keywords *w*_0_ and *w*_1_ trapdoor had not been queried by A. the simulator B generates keyword index as follows:

1. the simulator B first executes *H*_1_ queries twice to obtain $h_{0},h_{1}\in G_{0}$ such that *H*_1_(*w*_0_) = *h*_0_, *H*_1_(*w*_1_) = *h*_1_. For *i* = 0,1, we set (*w*_*i*_,*h*_*i*_,*e*_*i*_,*c*_*i*_) corresponding to the tuple on the *H*_1_-list. If both *c*_0_ = 0 and *c*_1_ = 1, then B declares the failure and aborts.
2. we known that at least one of *c*_0_ and *c*_1_ is equal to 0. The simulator B chooses a bit *b* ∈ {0,1} such that *c*_*b*_ = 0.
3. The simulator B answers the keyword index $I_{W}^{*}=(I_{1}^{*},I_{2}^{*})$. The simulator B then randomly chooses a parameter $t_{2}\in Z_{p}$ and sets I1*=u31t2 with the implied setting (ξi=ct2), where *c* is unknown, and we know *u*_3_ = *g*^*c*^ is a part of BDH.
4. the simulator B randomly chooses a *Z* ∈ {0,1}^*logp*^, and sets $I_{2}^{*}=Z$.

With the definition, $I_{W}^{*}=(I_{1}^{*},I_{2}^{*})$ is an effective keyword index for keyword *w*_*b*_ as queried.

**Phase 2**. Phase1 is repeated.

**Output**. The adversary A outputs its guess *b*′ of bit *b*.

Note the values $h_{i}=u_{2}^{e_{i}}$ be set with probability $\frac{1}{q_{T}+1}$ in the setting of the *H*_1_ queries. Since A queries the value of the form to *H*_2_ oracle

e((Bpub)ξi,H1(wb))=e((gat1)ct2,gbeb)=e(g,g)abc(ebt1t2)

with the same probability $\frac{1}{q_{T}+1}$ in the setting of the *H*_2_-list, therefore

(e(g,g)abc(ebt1t2),H2(e((Bpub)ξi,H1(wb))))∈H2

Then, the simulator B randomly chooses a pair (*t*_*i*_,*V*_*i*_) ∈ *H*_2_-list and outputs tebt1t2 as its guess for *e*(*g*,*g*)^*abc*^. Where *t*_1_,*t*_2_ and *e*_*b*_ are set according to the parameters of the challenge phase.

**Probability Analyses.** We can prove that A can win the game with a non negligible probability, then B can solve the BDH problem with the probability at least $\frac{2\varepsilon }{{{eq}_{T}q}_{H_{2}}}$. The specific probability analysis is similar to the scheme[34].

Because of the BDH assumption that the BDH problem is tough, so the probability $\frac{2\varepsilon }{{{eq}_{T}q}_{H_{2}}}$. is negligible. So that our scheme is secure under the BDH assumption.

## Computational complexity and performance evaluation

### Computational complexity comparison

In Table 3,we give the comparison of the computational complexity of our scheme with the schemes [13,19, 21,31]. As shown in Table 3, our scheme has a less amount of computation in the key generation and encryption generation compared with the schemes in [13,19,31]. Actually, our scheme needs the minimum amount of computation when the users decrypt the ciphertext. And most important is that our scheme need not update ciphertext when an attribute update occurs, which also help us greatly reduce the amount of computation. In addition, our scheme have the function of the keyword search, which can make the search more efficiently and more accurately. The schemes of [13],[19] and [21] don’t achieve the function of keyword search.

**Table 3:** Comparison of computational complexity.

schemes	[13]	[19]	[21]	[31]	ours
*PP*	(*m* + 4)*e* + *p*	(*m* + 2)*e* + *p*	(*m* + 2)*e* + *p*	(2*m* + 2)*e* + *p*	(2*m* + 3)*e* + *p*
*SK*	(2*l*_1_ + 11)*e*	(3*l*_1_ + 5)*e*	(*l*_1_ + 5)*e*	(3*l*_1_ + 2)*e*	(2*l*_1_ + 2)*e*
*CT*	(3*l*_2_ + 4)*e* + *p*	(3*l*_2_ + 4)*e* + *p*	(*l*_2_ + 4)*e* + *p*	(2*l*_2_ + 2)*e* + *p*	(3*l*_2_ + 2)*e* + *p*
*KeyUpdate*	*l*_3_*e*	(2*l*_3_ + 1)*e*	*l*_3_*e*	*l*_3_*e*	(*l*_3_ + 1)*e*
*CTUpdate*	*l*_4_*e*	*l*_4_*e*	*l*_4_*e*	*l*_4_*e*	✘
*Trapdoor*	✘	✘	✘	*e* + *p*	2*e*
*Index*	✘	✘	✘	*e* + 2*p*	*e* + 2*p*
*CS*. *Decrypt*	✘	✘	2*e* + (*l*_5_ + 2)*p*	✘	(*l*_5_ + 1)*p*
*U*ser. *Decrypt*	2*e* + (*l*_5_ + 2)*p*	4*e* + (*l*_5_ + 6)*p*	2*e* + *p*	2*e* + (1 + *l*_5_)*p*	2*e* + *p*

*e*:an exponential operation in $G_{0},G_{1}$

*p*: a pairing operation

*m*: the number of attributes in universe

*l*_1_: the number of attributes in the user's private key

*l*_2_: the number of attributes in the access structure

*l*_3_: the number of user attributes that need to be updated

*l*_4_: the number of attributes in the ciphertext that need to be updated

*l*_5_: the number of user's attributes satisfying an access control

✘: there is no corresponding function or process in the scheme.

### Performance evaluation

**Fig 2:**
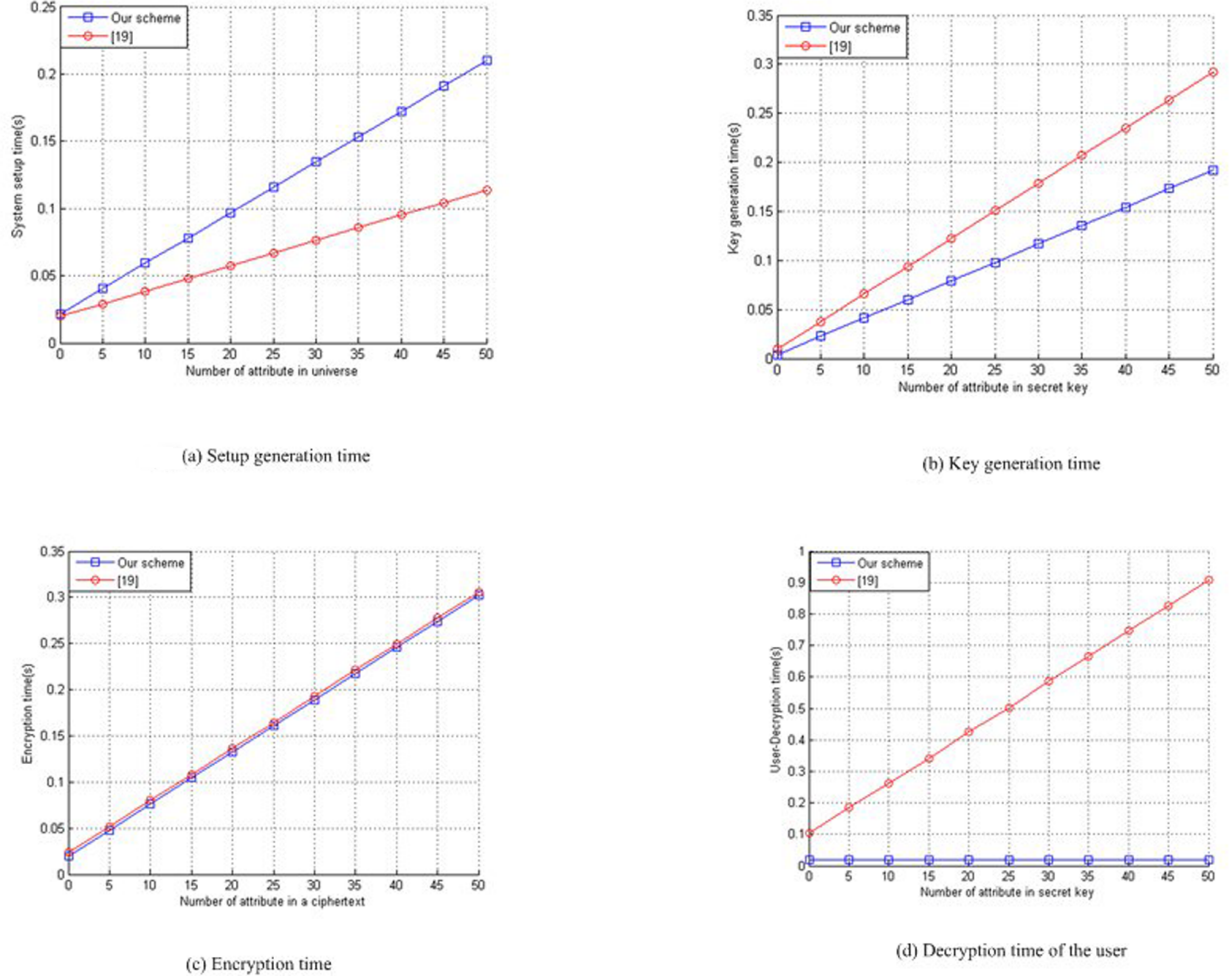
Performance evaluation. (a) Setup generation time (b) Key generation time (c) Encryption time (d) Decryption time of the user.

To evaluate the performance of our scheme and the scheme [19], we simulate the computational time of the setup generation, key generation, encryption and decryption by user with different number of attributes. As shown in Fig 2. The implementation is executed by using of the Pairing Cryptography (PBC) library[37]. We can clearly see from Fig 2(A) that the setup generation times scales linearly in the number of attribute in attribute universe in both scheme. Fig 2(B) shows secret key times scales linearly in the number of attribute in secret key in both scheme. Fig 2(C) shows the encryption times scales linearly in the number of attribute in ciphertexts in both scheme. The setup generation time is shown in Fig 2(A). We find also that the setup generation takes higher computational time in our scheme than the scheme [19]. The key generation time is shown in Fig 2(B) and the encryption time is shown in Fig 2(C). Obviously, the encryption time and key generation time of the scheme[19] takes higher computational time than our scheme.Fig 2(D) shows that the user- decryption time of our scheme takes lesser computational time than the scheme [19].

## Conclusions

In this paper, we have proposed a keyword searchable attribute-based encryption scheme with attribute update for cloud storage. Our new scheme supports both the user's attribute update and supports multi-user keywords search, as long as user's trapdoor could match keyword index stored in the cloud storage, then the user can search interesting encrypted file successfully. The performance evaluation results confirm that the proposed scheme is more efficient than other attribute based encryption schemes with attribute update. In addition, we outsource the operation with high computation cost to the cloud storage to reduce the user's computational burden. Moreover, our scheme also is proven to be semantic security against chosen ciphertext-policy and chosen plaintext attack in the general bilinear group model.

## Supporting information

S1 File (ZIP)
