# Supplementary material for: A keyword searchable attribute-based encryption scheme with attribute update for cloud storage
Source: PLoS One. 2018 May 24;13(5):e0197318. doi: 10.1371/journal.pone.0197318 (PMC5967729; doi:10.1371/journal.pone.0197318)
Supplement: S1 File — (ZIP) [file pone.0197318.s001.zip › S1 Supporting Information .docx]

**S1  Supporting Information**

The experimental part of this paper refers to the results in the Pairing Based Cryptography (PBC) library.

**Table .The runtime of cryptographic operations**

| Operating | Ad | Ne | Mu | In | Ex | Add | Neg | PM | Mul | Inv | Exp | P |
| --- | --- | --- | --- | --- | --- | --- | --- | --- | --- | --- | --- | --- |
| Times/ms | 0.001 | 0.000 | 0.001 | 0.004 | 0.067 | 0.038 | 0.001 | 8.006 | 0.013 | 0.041 | 1.882 | 16.064 |

^1^Ad: an addition operation in $\mathbb{Z}_{P}$;

^2^Ne: the inverse in addition operation in $\mathbb{Z}_{P}$;

^3^Mu: a multiplication operation in $\mathbb{Z}_{P}$;

^4^In: the inverse in multiplication operation in $\mathbb{Z}_{P}$;

^5^Ex: an exponentiation operation in $\mathbb{Z}_{P}$;

^6^Add: an addition operation in $\mathbb{G}_{0}$;

^7^Neg: the inverse in addition operation in $\mathbb{G}_{0}$;

^8^PM: a point multiplication operation in $\mathbb{G}_{0}$;

^9^Mul: a multiplication operation in $\mathbb{G}_{1}$;

^10^Inv: the inverse in multiplication operation in $\mathbb{G}_{1}$;

^11^Exp: an exponentiation operation in $\mathbb{G}_{1}$;

^12^P: an bilinear pairings operation in $\mathbb{G}_{1}$.
